# Utility of the Amygdalar Atrophy Scale to Identify Patients With Limbic-Predominant Age-Related TDP-43 Encephalopathy in a Memory Clinic

**DOI:** 10.1212/WNL.0000000000214477

**Published:** 2025-12-22

**Authors:** Fausto Roveta, Francesca B. Pizzini, Valerio Natale, Max Scheffler, Valentina Garibotto, Augusto J. Mendes, Chen Wang, Aurelien Lathuiliere, Christian Chicherio, Innocenzo Rainero, Giovanni B. Frisoni, Federica Ribaldi

**Affiliations:** 1 Aging Brain and Memory Clinic, Department of Neuroscience “Rita Levi-Montalcini”, University of Turin, Italy;; 2 Geneva Memory Center, Department of Rehabilitation and Geriatrics, Geneva University Hospitals, Switzerland;; 3 Laboratory of Neuroimaging of Aging (LANVIE), University of Geneva, Switzerland;; 4 Department of Engineering for Innovation Medicine, University of Verona, Italy;; 5 Radiology, Department of Diagnostic and Public Health, University of Verona, Italy;; 6 Division of Radiology, Geneva University Hospitals, Switzerland;; 7 Division of Nuclear Medicine and Molecular Imaging, Geneva University Hospitals, Switzerland;; 8 Laboratory of Neuroimaging and Innovative Molecular Tracers (NIMTlab), Geneva University Neurocenter and Faculty of Medicine, University of Geneva, Switzerland;; 9 CIBM Center for Biomedical Imaging, Geneva, Switzerland; and; 10 Laboratory for translational research in neurodegeneration, University of Geneva, Switzerland.

## Abstract

**Background and Objectives:**

Limbic-predominant age-related TDP-43 encephalopathy (LATE) is a neurodegenerative condition often overlapping clinically with Alzheimer disease (AD). No established in vivo biomarkers exist for LATE. The amygdalar atrophy scale (AAS) is a visual MRI rating tool scoring the amygdala as AAS_0_ (no atrophy), AAS_1_ (mild-to-moderate atrophy), or AAS_2_ (severe atrophy) and is associated with TDP-43 neuropathology in limbic regions. This study describes the clinical, neuroimaging, and longitudinal cognitive profiles of individuals classified using a proposed framework that combines the AAS with established AD biomarkers.

**Methods:**

This retrospective study included individuals attending the Geneva Memory Center (2014–2022) who underwent T1-weighted MRI, as well as clinical and neuropsychological assessments. Participants were categorized with an etiologic grouping defined as No-AD/No-LATE (AAS_0_, negative AD biomarkers), AD (AAS_0_, positive AD biomarkers), LATE (AAS_1–2_, negative AD biomarkers), or AD/LATE (AAS_1–2_, positive AD biomarkers). Group differences were compared using Kruskal-Wallis or χ^2^ tests. Differences in MRI brain volumes and cortical thickness between groups were examined with analysis of covariance. Cognitive trajectories using Mini-Mental State Examination (MMSE) scores over 30 months were estimated with linear mixed-effects models accounting for baseline age and education.

**Results:**

The analytic sample included 469 participants (mean age 71.2 ± 8.5 years, 53% female). The No-AD/No-LATE group exhibited the highest MMSE scores (N = 181, 27.9 ± 2.2), outperforming AD (N = 146, 24.2 ± 4.9), LATE (N = 36, 26.2 ± 3.2), and AD/LATE (N = 106, 22.5 ± 5.3) groups (*p* < 0.001). On the Free and Cued Selective Reminding Test, the No-AD/No-LATE group showed the highest scores (15.1 ± 1.8); the LATE and AD groups performed similarly (13.3 ± 3.5 and 12.5 ± 4.0), both exceeding the AD/LATE group (10.6 ± 4.1) (*p* < 0.001). LATE and AD/LATE groups showed lower volumes/thicknesses in TDP-43–related regions compared with AD and No-AD/No-LATE groups. Longitudinally, the LATE group maintained cognitive stability as the No-AD/No-LATE group (β = −0.010, 95% CI −0.096 to 0.075, *p* = 0.813), whereas both AD (β = −0.136, 95% CI −0.184 to −0.088, *p* < 0.001) and AD/LATE (β = −0.139, 95% CI −0.196 to −0.082, *p* < 0.001) groups showed faster decline over time.

**Discussion:**

Patients identified as LATE by the integration of AAS with AD biomarkers had generally mild amnestic cognitive impairment, significant limbic atrophy, and slow decline over time, all features consistent with previous autopsy series. Moreover, AD/LATE cases were more impaired than those with LATE or AD alone. Prospective and neuropathologic validation is warranted.

## Introduction

Limbic-predominant age-related TDP-43 encephalopathy (LATE) is a prevalent yet under-recognized neurodegenerative disease, particularly in the aging population, where it represents a significant contributor to cognitive decline.^1,2^ Patients with a postmortem diagnosis of LATE are typically older and present an amnestic cognitive syndrome that is often challenging to clinically differentiate from other causes, including Alzheimer disease (AD). However, longitudinal assessment may aid in differentiation because LATE is characterized by a slower rate of cognitive decline compared with AD.^3,4^ Recognition of LATE as a distinct clinicopathologic entity is relatively recent. Indeed, as of today, there are no validated in vivo biomarkers of LATE neuropathologic changes (LATE-NCs), although some are emerging.^5-7^ By contrast, the field of biomarker-based in vivo diagnosis of AD neuropathologic changes (ADNCs) is well established and rapidly evolving.^8,9^

LATE-NC is characterized by the accumulation of phosphorylated transactive response DNA-binding protein 43 kDa (TDP-43) within the limbic system.^10,11^ Neuropathologic studies have shown that the earliest sites of TDP-43 accumulation include the amygdala, followed by the hippocampus and the entorhinal cortex.^5^ In addition to mesial temporal lobe atrophy, LATE-NC is associated with atrophy in the inferior frontal, anterior temporal, and insular cortices.^12^ This regional atrophy pattern closely aligns with the distribution of TDP-43 pathologic protein spread observed at autopsy.^13^ Complicating matters further, ADNC and LATE-NC frequently coexist within the same brain, highlighting the urgent need to develop diagnostic methods capable of distinguishing the 2 entities to enable accurate prognosis and timely and targeted therapeutic intervention.^3,5^

Provisional diagnostic criteria have been recently proposed for identifying probable or possible LATE, either in its isolated form or in comorbidity with AD.^6^ However, the authors of these criteria acknowledge several areas requiring further research, including the development of reliable TDP-43 biomarkers and neuroimaging tools to facilitate the clinical diagnosis of LATE.

The amygdala, the earliest site affected by LATE-NC, has been widely recognized as being associated with volume reduction and morphological changes linked to TDP-43 pathology.^14,15^ It is important to note that the amygdala is also an early site of accumulation of tau neurofibrillary tangles in ADNC, as established by Braak and Braak decades ago.^16^ Advances in imaging analysis techniques have renewed interest in studying the role of amygdala in neurodegenerative diseases.^17,18^ Recently, a new amygdalar atrophy scale (AAS) has been introduced as a reliable and valid tool for assessing amygdalar atrophy using MRI.^19^ This visual, MRI-based rating system categorizes amygdalar atrophy into 3 levels: AAS_0_ (no atrophy), AAS_1_ (mild-to-moderate atrophy), and AAS_2_ (severe atrophy). The AAS demonstrated high inter-rater reliability and provides complementary, nonredundant information to the well-established medial temporal atrophy (MTA) scale.^19^ In addition, the AAS has shown a strong correlation with automated measurements of amygdalar volume and exhibits a linear association with LATE-NC presence in medial temporal lobe structures, as validated in an autopsy series of nearly 100 participants.^19^

The primary aim of this study was to characterize the clinical and neuroimaging features and longitudinal cognitive trajectories of individuals classified through a proposed biomarker-based approach that integrates the AAS with established AD biomarkers, thereby assessing the potential utility of the AAS in memory clinics for identifying probable cases of LATE. By combining the AAS with biomarkers for AD, we aim to (1) describe the demographic, clinical, and neuropsychological profiles of the identified participants; (2) examine neuroimaging correlates by analyzing cortical volumes and thickness in brain regions associated with both LATE and AD; and (3) characterize longitudinal cognitive trajectories to evaluate the rate of cognitive decline in the included participants.

## Methods

### Population

We retrospectively selected all patients with at least 1 available MRI scan consulting the Geneva Memory Center (GMC) of the Geneva University Hospitals from 2014 to 2022. The GMC cohort includes individuals ranging from cognitively unimpaired (CU) participants, either healthy volunteers or individuals with subjective cognitive decline (SCD), to patients with mild cognitive impairment (MCI) and dementia. The inclusion criteria for this study were as follows: (1) availability of valid T1-weighted MRI to perform AAS scoring and (2) availability of at least 1 clinical and 1 neuropsychological evaluation. Exclusion criteria were as follows: (1) ongoing or unresolved diagnosis and (2) diagnosis of major psychiatric disorders. Each participant had thus already undergone clinical and neuropsychological evaluation as part of the standard workup at the memory clinic. Cognitive tests were available for patients if it was clinically deemed or conducted in the context of research studies. We reported the tests for which availability was at least 50%. Neuropsychological standard tests included assessment of global cognition (Mini-Mental State Examination [MMSE], Three-Objects-Three-Places, Clock Drawing Test). Additional tests assessed episodic memory (Free and Cued Selective Reminding Test [FCSRT] with delayed recall), working memory (digit span forward and backward), executive functions (Trail Making Test B, category and phonemic fluency), and processing speed (Trail Making Test A). Diagnostic classification followed established criteria described elsewhere.^20^ In brief, SCD was defined as self-reported cognitive deterioration without objective impairment on neuropsychological testing. MCI was defined by objective cognitive deficits, cognitive concerns reported by the patient or informant, and preserved daily functioning. Dementia was diagnosed in individuals who met criteria for cognitive impairment but also exhibited impairment in activities of daily living. Subgroups of participants also underwent PET scans with tracers for amyloid or tau pathologies. These additional investigations were performed when clinically indicated or as part of research studies. Available CSF analyses for the assessment of β-amyloid (Aβ) 42/40 and phosphorylated tau 181 (p-tau181) levels were also considered. Only PET or CSF biomarkers obtained within 1 year of the MRI acquisition were included in the analyses. Information on APOE genotype was collected for participants when available. In addition, a subset of participants underwent follow-up neuropsychological evaluations, with the duration and number of follow-up visits varying based on clinical or research requirements. We followed the Strengthening the Reporting of Observational Studies in Epidemiology checklist when writing our report.^21^

### Imaging and Biological Sample Protocols

#### MRI

MRI scans were acquired using a 3T MRI scanner (Magnetom Skyra, Siemens Healthineers, Nuremberg, Germany) at the Division of Radiology at Geneva University Hospitals, following a previously established protocol.^19^ Three trained raters (F.B.P., V.N., M.S.) independently assessed T1-weighted MRI scans to assign AAS scores, as detailed in a previous study.^19^ MTA scores were available for 1,439 participants and were rated using the 5-point MTA scale, with the average score of the left and right sides used for analysis.^22^ Brain volumes and cortical thicknesses were extracted using FreeSurfer (version 7.3.2 recon-all).^23^ Regions of interest were selected to represent areas known to be affected in earlier stages of TDP-43 deposition, as defined by proposed neuropathologic classifications.^5,13^ These included the amygdala, hippocampus, entorhinal cortex, temporal pole, and insula. In addition, the thicknesses of the precuneus and posterior cingulate cortex were evaluated because these regions are known to be associated with ADNC.^13^ For both volumes and cortical thicknesses, we used the mean of right and left hemisphere measures. Volumes were standardized for total intracranial volume (TIV) using the formula (volume × mean population TIV)/(single-participant TIV).

#### PET

The amyloid-PET images were acquired using ^18^F-florbetapir or ^18^F-flutemetamol tracers while tau-PET images were acquired using ^18^F-flortaucipir following a previously described protocol.^24^ Acquisitions were performed on Siemens Biograph and Biograph Vision scanners (Siemens, Washington, DC) and reconstructed using 3D OSEM iterative reconstruction, with corrections for randoms, dead time, normalization, scatter, attenuation, and sensitivity. Amyloid positivity and tau Braak stages were visually assessed by an expert nuclear medicine physician (V.G.), following the standard operating procedures approved by the European Medicines Agency^25^ and published recommendations.^26^ For tau-PET imaging, in particular, we adapted the visual classification to further classify negative participants as Braak 0, showing no visible uptake in cortical regions, or Braak I–III, when showing uptake limited to the medial temporal areas.^27^

#### Biological Samples

CSF and blood samples were collected following GMC standardized procedures previously described. In brief, CSF was obtained by lumbar puncture with an atraumatic needle after an overnight fast, gathered in polypropylene tubes, centrifuged within an hour at 2,000*g* for 10 minutes at room temperature, then aliquoted into polypropylene vials, and frozen at −80°C. Levels of Aβ40, Aβ42, and p-tau181 in CSF were measured using the LumiPulse G600II instrument with commercial kits #230336, 231524, and 230350 (Fujirebio Europe, Ghent, Belgium) at a clinical chemistry laboratory of Geneva University Hospitals. Following the AT(N) classification system, the Aβ42/40 ratio was used for establishing “A” positivity, while p-tau181 was used for “T” positivity based on clinically CSF cohort-specific thresholds (Aβ42/40 <0.09 and p-tau181 >86.2).^28^ Plasma was collected in EDTA tubes, kept for 2 hours at room temperature before centrifugation (1,700*g*, 15 minutes), aliquoted as 500 μL in 1.2-mL polypropylene tubes, and stored at −80°C in the local biobank. DNA was extracted from the remaining blood in the plasma EDTA tubes using the automated QIAsymphony DSP DNA Kit #937255 (Qiagen, Venlo, Netherlands). APOE genotyping was performed using TaqMan genotyping MasterMix assay #4371355 (Thermo Fisher, Waltham, MA).

### Classification of Participants Into Etiologic Diagnostic Groups

Patients were classified according to their biomarker status, based on either established AD biomarkers or the AAS score as a potential LATE biomarker. In fact, for the etiologic diagnostic groups, only individuals for whom at least 1 valid AD biomarker, either PET or CSF, was available were included. Cases in which the biomarker profile was uncertain or discordant regarding the probable biological etiology (cases with negative amyloid-PET but positive tau-PET, or cases with a CSF profile of A+/T− or A−/T+) were excluded a priori from the etiologic classification. In cases where multiple AD biomarkers were available, priority was given to amyloid-PET, followed by CSF A/T status over tau-PET Braak stage to classify cases. Tau-PET imaging cases showing isolated uptake in the mesial temporal cortex (Braak I–III) without amyloid status information were excluded, as the possibility of ADNC, or more rarely primary age-related tauopathy or other etiologies, could not be definitively ruled out.

To identify the probable isolated ADNC-positive biomarker cases (hereafter referred as “AD”), an AAS score of 0 (AAS_0_) was required in combination with at least 1 positive AD biomarker. When CSF or PET amyloid status was unavailable, a positive tau-PET (visual Braak stages IV–VI) was considered indicative of probable ADNC, as previously suggested.^29^ To identify patients with probable LATE-NC biomarker cases (hereafter referred as “LATE”), an AAS score >0 (AAS_1–2_) was required, along with the exclusion of AD pathology, defined as negative amyloid-PET, CSF A−/T−, or tau-PET Braak 0. Cases meeting the criteria for ADNC but with AAS_1–2_ were considered indicative of possible co-pathology involving AD and LATE (hereafter referred to as “AD/LATE”). Finally, cases with a negative AD biomarker profile and an AAS score of 0 were classified as probable individuals without ADNC or LATE-NC and designated as the “No-AD/No-LATE” group.

### Statistical Analysis

Statistical analyses were performed using SPSS version 29.0.1.0 and R Studio version 2024.09.0+375. Baseline differences in demographics, clinical features, and cognitive performance across etiologic diagnostic groups (No-AD/No-LATE, AD, LATE, AD/LATE) were assessed using the Kruskal-Wallis test for continuous variables and the χ^2^ test for categorical variables. Post hoc pairwise differences were evaluated using the Mann-Whitney *U* test with Bonferroni correction, and post hoc comparisons for categorical variables were performed using *z*-tests for 2 proportions with Bonferroni correction for multiple comparisons. To test whether considering only a symptomatic cohort would confirm the findings, the tests were repeated after excluding the CU subgroup as sensitivity analysis. A partial correlation analysis was performed to evaluate the relationship between AAS scores and age, while accounting for differences in cognitive stage distribution. Analysis of covariance with age of participants as a covariate was conducted to assess differences in MRI volumes and cortical thicknesses in selected brain regions, with the Tukey honestly significant difference post hoc test used for pairwise comparisons. To analyze cognitive decline over time across etiologic diagnostic groups, a linear mixed-effects (LME) model was used to estimate cognitive trajectories over a period of 30 months. Fixed effects included baseline age, years of education, etiologic diagnostic group, time (in months), and the interaction between time and etiologic diagnostic group (time × diagnostic group). Random intercepts and slopes by participant were incorporated to account for individual variability. The model was fitted using restricted maximum likelihood estimation, and *p* values were calculated using the Satterthwaite approximation for degrees of freedom. Pairwise comparisons among the etiologic diagnostic groups at 30 months were conducted using the estimated marginal means of the MMSE score, thereby allowing estimation of the ΔMMSE score among the 4 groups. Tukey adjustment was applied to account for multiple comparisons. Statistical significance was defined as *p* < 0.05.

### Standard Protocol Approvals, Registrations, and Participant Consents

The study was approved by the Regional Research Ethics Committee (PB_2016-01346, 2020_00403) and conducted in accordance with the principles of the Declaration of Helsinki and the International Conference on Harmonization Good Clinical Practice. All participants gave their written informed consent to participate in this study.

### Data Availability

Anonymized data not published within this article will be made available by request from any qualified investigator to the corresponding author.

## Results

The original cohort comprised 1,653 participants. Their key demographic and clinical characteristics are given in eTable 1. AAS scores showed a moderate correlation with age (ρ = 0.41, *p* < 0.001), consistent across cognitive stages.

Considering participants with at least 1 valid AD biomarker assessed within 1 year of MRI, the final analytic cohort comprised 469 individuals, whose key demographic, clinical, and biomarker characteristics are presented in Table 1.

**Table 1:** Demographics, Cognitive Features, and Biomarker Status of the Analytic Cohort by Cognitive Stages

Variable	CU (N = 127)	Missing (%)	MCI (N = 240)	Missing (%)	Dementia (N = 102)	Missing (%)
Demographics						
Age, y	66.8 (8.8)	—	72.7 (7.7)	—	73.2 (7.8)	—
Sex, male (%)	55 (43)	—	115 (48)	—	50 (49)	—
Years of education	15.4 (3.8)	—	13.7 (3.9)	—	12.0 (3.9)	—
Cognitive features						
Mini-Mental State Examination	28.8 (1.1)	7.9	26.2 (2.7)	2.9	19.6 (5.5)	2.9
Clock Drawing Test	9.5 (0.9)	7.9	8.2 (2.1)	3.8	5.9 (2.7)	8.8
Three-Objects-Three-Places	8.9 (0.5)	27.6	7.0 (2.3)	18.3	3.8 (3.0)	27.5
Digit span	25.8 (5.2)	15.0	21.1 (4.8)	20.8	15.7 (4.4)	39.2
FCSRT delayed free recall	12.6 (2.2)	15.0	6.6 (4.1)	28.3	2.9 (3.0)	72.5
FCSRT delayed total recall	15.8 (0.6)	15.0	13.0 (3.2)	28.3	8.4 (4.5)	74.5
Verbal fluency (categorical)	21.2 (5.4)	25.2	15.3 (5.0)	27.5	9.3 (3.5)	48.0
Verbal fluency (phonemic)	21.6 (6.9)	26.0	15.3 (6.4)	30.4	9.2 (5.9)	52.9
Trail Making Test A	39.4 (17.2)	13.4	56.1 (30.2)	24.6	106.7 (87.4)	60.8
Trail Making Test B	91.2 (40.3)	11.8	146.3 (89.5)	31.7	178.8 (91.4)	84.3
Biomarkers						
AAS_0_/AAS_1_/AAS_2_ (%)	96.1/3.9/0.0	—	67.5/30.4/2.1	—	42.2/52.9/4.9	—
Mean MTA scale score	0.63 (0.67)	17.3	1.18 (0.84)	5.4	1.69 (0.84)	9.8
Amyloid-PET positivity (%)	21 (19.6)	15.7	123 (64.7)	20.8	68 (86.1)	22.5
Tau-PET positivity (%)	5 (8.2)	52.0	44 (40.7)	55.0	26 (81.3)	68.6
CSF Aβ42/40	0.095 (0.023)	91.3	0.085 (0.033)	89.2	0.055 (0.022)	83.3
CSF p-tau181	45.6 (46.0)	91.3	64.8 (46.5)	89.2	121.4 (64.9)	83.3
Genetic status						
APOE (ε4 carrier, %)	52 (27)	18.1	81 (44)	42.1	26 (57)	63.7

Abbreviations: AAS = amygdalar atrophy scale; Aβ = β-amyloid; CU = cognitively unimpaired; FCSRT = Free and Cued Selective Reminding Test; MCI = mild cognitive impairment; MTA = medial temporal lobe atrophy; p-tau181 = phosphorylated tau 181.

Numerical data are presented as the mean (SD) while categorical data are expressed as the number (percentage of the total).

### Clinical Characteristics of Etiologic Diagnostic Groups

**Figure 1:**
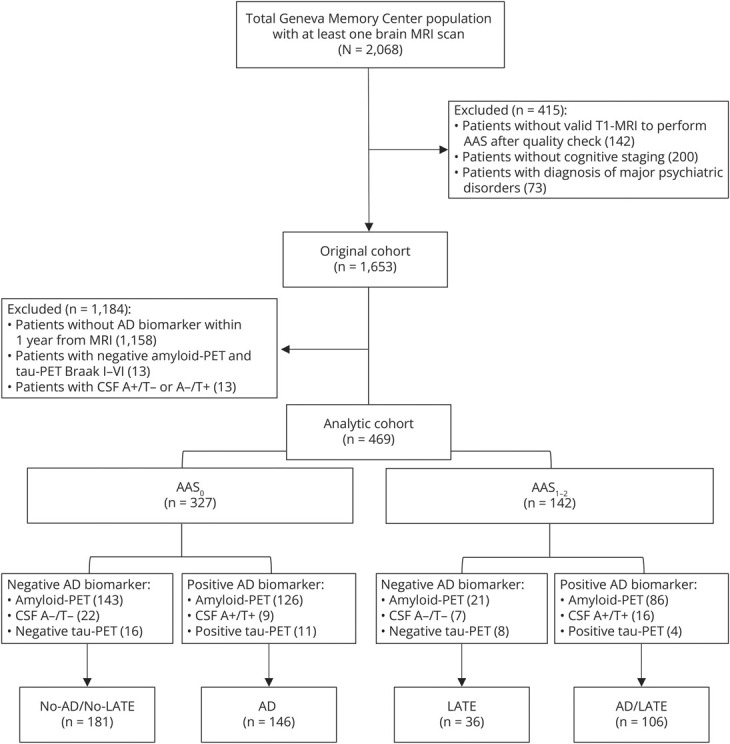
Flowchart for Inclusion/Exclusion and Classification of Participants Into Etiologic Diagnostic Groups AAS = amygdalar atrophy scale; AD = Alzheimer disease; AD/LATE = AD and LATE co-pathology; LATE = limbic-predominant age-related TDP-43 encephalopathy.

Combining AAS scores and AD biomarkers, 4 diagnostic etiologies were identified: No-AD/No-LATE (N = 181), AD (N = 146), LATE (N = 36), and AD/LATE (N = 106). A flowchart illustrating the application of primary inclusion and exclusion criteria, as well as the classification of participants into final diagnostic groups, is presented in Figure 1. Demographic and clinical information, neuropsychological features, and APOE ε4 carriership of patients categorized by these etiologic diagnostic groups are given in Table 2.

**Table 2:** Demographics, Cognitive Stages, Cognitive Features, and Genetic Status of Participants by Etiologic Diagnostic Groups

Variable	No-AD/No-LATE(N = 181)	Missing (%)	AD(N = 146)	Missing (%)	LATE(N = 36)	Missing (%)	AD/LATE(N = 106)	Missing (%)	*p* Value
Demographics									
Age, y	67.5 (8.8)^c^	—	71.5 (8.5)^b^	—	75.0 (6.5)^a^	—	75.5 (5.6)^a^	—	<0.001
Sex, male (%)	83 (46)^b^	—	60 (41)^b^	—	24 (67)^a^	—	54 (51)	—	0.031
Years of education	14.4 (3.78)	—	13.4 (4.4)	—	13.1 (4.5)	—	13.3 (3.6)	—	0.157
Cognitive stages									
CU/MCI/dementia, %	55/42^b^/3	—	16/58^a^/26	—	8/67^a^/25	—	2/51/47	—	<0.001
Cognitive features									
Mini-Mental State Examination	27.9 (2.15)^a^	4.4	24.2 (4.9)^b^	6.2	26.2 (3.2)^b^	—	22.5 (5.3)^c^	1.9	<0.001
Clock Drawing Test	9.0 (1.6)^a^	5.0	7.6 (2.6)^b^	8.2	8.1 (1.7)^b^	—	7.1 (2.8)^b^	5.7	<0.001
Three-Objects-Three-Places	8.4 (1.5)^a^	25.4	5.9 (2.8)^b^	18.5	8.0 (2.1)^a^	22.2	5.1 (3.0)^b^	24.5	<0.001
Digit span	23.6 (5.6)^a^	12.7	20.4 (6.0)^b^	30.8	21.6 (5.3)	22.2	18.9 (5.1)^b^	30.2	<0.001
FCSRT delayed free recall	10.6 (3.8)^a^	14.9	7.0 (4.8)^b^	45.9	6.8 (4.1)^b^	22.2	4.3 (3.8)^c^	54.7	<0.001
FCSRT delayed total recall	15.1 (1.8)^a^	14.9	12.5 (4.0)^b^	45.9	13.3 (3.5)^b^	22.2	10.6 (4.1)^c^	54.7	<0.001
Verbal fluency (categorical)	19.1 (6.1)^a^	28.2	15.1 (5.5)^b^	37.7	14.3 (4.8)^b^	19.4	12.3 (6.0)^c^	32.1	<0.001
Verbal fluency (phonemic)	18.6 (7.6)^a^	28.7	15.8 (7.5)	41.8	14.7 (8.0)	22.2	13.1 (6.3)^b^	36.8	<0.001
Trail Making Test A	43.4 (21.2)^b^	16.6	61.4 (47.3)^a^	38.4	67.7 (59.4)^a^	30.6	75.2 (58.3)^a^	37.7	<0.001
Trail Making Test B	104.1 (71.0)^c^	18.8	151.4 (87.8)^a^	47.3	127.4 (63.7)^b^	41.7	156.1 (78.3)^a^	53.8	<0.001
Genetic status									
APOE (ε4 carriers)	34 (25)^b^	24.9	46 (61)^a^	47.9	4 (21)^b^	47.2	27 (55)^a^	53.8	<0.001

Abbreviations: AD = Alzheimer disease; AD/LATE = AD and LATE co-pathology; CU = cognitively unimpaired; FCSRT = Free and Cued Selective Reminding Test; LATE = limbic-predominant age-related TDP-43 encephalopathy; MCI = mild cognitive impairment.

Numerical data are presented as the mean (SD) while categorical data are expressed as the number (percentage of the total). Post hoc comparisons are reported as a > b > c > d.

A significant difference in age was observed across etiologic diagnostic groups (*H*(3) = 73.888, *p* < 0.001). Participants with LATE and AD/LATE were older (75.0 ± 6.5 years and 75.5 ± 5.6 years, respectively) compared with those in the AD group (71.5 ± 8.5 years). Male participants were more frequent in the LATE group (67%) compared with the No-AD/No-LATE (46%) and AD (41%) groups (*p* = 0.031).

Cognitive stage was significantly associated with diagnostic groups (χ^2^ = 156.054, *df* = 6, *p* < 0.001). Post hoc tests showed higher MCI prevalence in the LATE (67%) and AD (58%) groups compared with the No-AD/No-LATE group (42%) (*p* < 0.001).

Mean MMSE scores were highest in the No-AD/No-LATE group (27.9 ± 2.15), followed by the LATE (26.2 ± 3.2) and AD (24.2 ± 4.9) groups, which did not differ significantly from each other. All 3 groups scored significantly higher than the AD/LATE group, which had the lowest MMSE scores (22.5 ± 5.3; *H*(3) = 135.801, *p* < 0.001). On the Three-Objects-Three-Places test, the No-AD/No-LATE and LATE groups performed similarly (8.4 ± 1.5 and 8.0 ± 2.1, respectively), both outperforming the AD (5.9 ± 2.8) and AD/LATE (5.1 ± 3.0) groups (*H*(3) = 108.193, *p* < 0.001).

For FCSRT delayed total recall, the No-AD/No-LATE group scored highest (15.1 ± 1.8), followed by similar performance in the LATE (13.3 ± 3.5) and AD (12.5 ± 4.0) groups, while the AD/LATE group had the lowest scores (10.6 ± 4.1; *H*(3) = 77.406, *p* < 0.001).

The sensitivity analysis including only cognitively impaired participants revealed findings consistent with the main results, as presented in eTable 2.

### MRI Measures of Etiologic Diagnostic Groups

Significant differences were observed among diagnostic groups in volumes and cortical thickness of regions associated with TDP-43 pathology.

Amygdalar (*F*(3,463) = 83.08, *p* < 0.001) and hippocampal (*F*(3,463) = 101.56, *p* < 0.001) volumes differed significantly across groups: the LATE and AD/LATE groups demonstrated greater atrophy compared with both AD and No-AD/No-LATE groups. Thickness in the entorhinal cortex (*F*(3,463) = 60.36, *p* < 0.001), temporal pole (*F*(3,463) = 50.80, *p* < 0.001), and insula (*F*(3,463) = 35.16, *p* < 0.001) also showed significant group differences.

**Figure 2:**
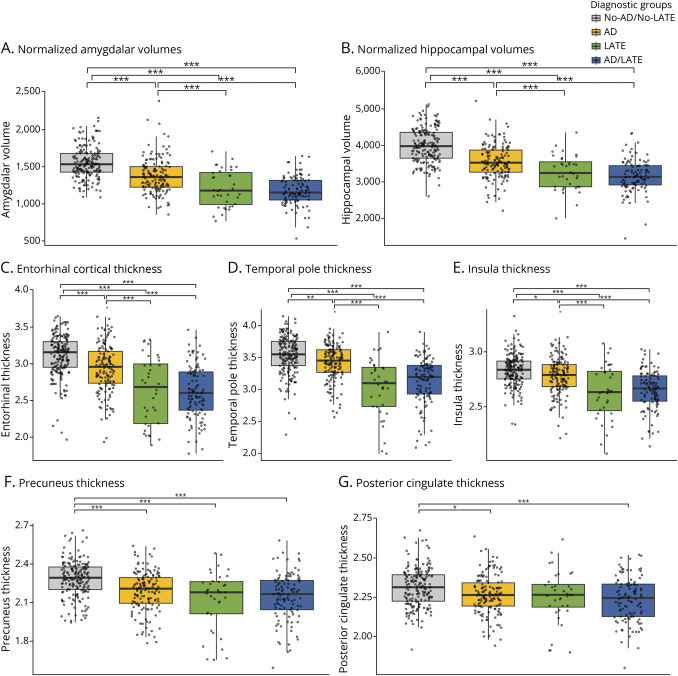
Boxplots of Volumes and Thicknesses in Brain Regions Associated With TDP-43 and Alzheimer Pathology by Etiologic Diagnostic Groups Boxplots (A–G) illustrate key differences in brain volumes and thicknesses across diagnostic groups. Panels (A) and (B) show significantly lower normalized volumes of the hippocampus and amygdala in the AD/LATE and LATE groups compared with the AD group. Panels (C–E) depict brain regions associated with TDP-43 deposition, revealing significantly reduced thickness in the AD/LATE and LATE groups. By contrast, panels (F) and (G) highlight regions associated with AD deposition, specifically the precuneus and posterior cingulate thicknesses, which exhibit no statistically significant differences among the AD, LATE, and AD/LATE groups. Volumes and thicknesses in all regions were reduced in AD, LATE, and AD/LATE groups compared with the No-AD/No-LATE group. The horizontal lines within the boxplots represent the median values, and the boxes illustrate the interquartile range (IQR). Individual data points (jittered) reflect the normalized volumes or thicknesses for each participant. AD = Alzheimer disease; AD/LATE = AD and LATE co-pathology; LATE = limbic-predominant age-related TDP-43 encephalopathy. Statistical significance is reported as ***p < 0.001, **p < 0.01, and *p < 0.05.

By contrast, differences were less marked in the precuneus (*F*(3,463) = 23.48, *p* < 0.001) and posterior cingulate cortex (*F*(3,463) = 8.59, *p* < 0.001), mainly reflecting higher cortical thickness in the No-AD/No-LATE group, with no significant differences among the other groups. Figure 2 presents boxplot representation of these findings.

### Cognitive Trajectories of Etiologic Diagnostic Groups

A total of 276 participants had at least 1 follow-up visit and were thus included in the LME analysis. The average follow-up period of the participants included in the LME model was 20.16 ± 13.68 months. Baseline demographics, cognitive stages, and cognitive features of participants included in the LME model are given in eTable 3.

**Figure 3:**
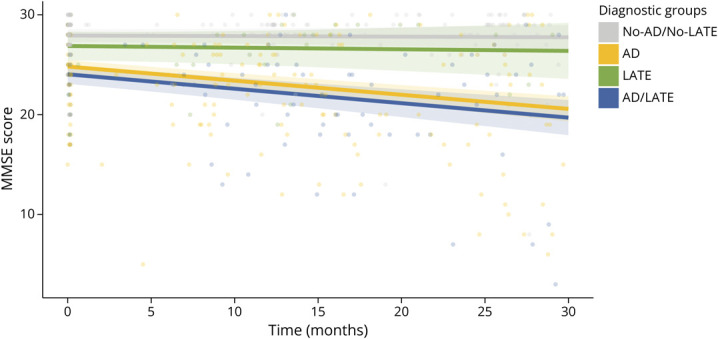
Cognitive Longitudinal Trajectories by Etiologic Diagnostic Groups Lines represent predicted MMSE trajectories from linear mixed-effects models including random intercepts and slopes for time by participant, with shaded areas indicating 95% CIs. Raw data points are overlaid to illustrate the coverage of observed values across follow-up. AD = Alzheimer disease; AD/LATE = AD and LATE co-pathology; LATE = limbic-predominant age-related TDP43 encephalopathy.

Over time, significant interactions were observed for the AD (β = −0.136, 95% CI −0.184 to −0.088, *p* < 0.001) and AD/LATE (β = −0.139, 95% CI −0.196 to −0.082, *p* < 0.001) groups, indicating faster cognitive decline compared with the No-AD/No-LATE group, whereas the LATE group showed a similar trajectory (β = −0.010, 95% CI −0.096 to 0.075, *p* = 0.813). Cognitive trajectories over time by the 4 diagnostic groups are presented in Figure 3.

At 30 months, pairwise comparisons showed greater MMSE score decline in the AD (ΔMMSE score = −5.797, *p* = 0.002) and AD/LATE (ΔMMSE score = −6.678, *p* = 0.001) groups vs the LATE group, with no significant difference between AD and AD/LATE (ΔMMSE score = 0.881, *p* = 0.804) groups. eTable 4 provides a complete summary of the LME model. The sensitivity analysis conducted after excluding CU participants confirmed similar results, as presented in eTable 5.

## Discussion

This study highlights the potential of the AAS as a novel, simple, and practical visual tool for identifying participants with probable LATE in memory clinic workups. By integrating AAS with established AD biomarkers, we stratified patients into distinct etiologic diagnostic groups. Participants classified as having No-AD/No-LATE, AD, LATE, or AD/LATE displayed different demographic, cognitive, and neuroimaging measures, as well as different cognitive trajectories.

LATE predominantly affects older individuals, often exceeding the age range of patients with other neurodegenerative diseases, including AD.^1^ In our study, AAS scores showed a significant moderate correlation with age and patients classified as LATE were significantly older than those classified as AD. These findings align with previous reports from autopsy series of LATE.^5,30^ Consistent with previous research, we observed a higher proportion of men in the LATE group compared with the AD group, suggesting potential sex-related differences in the risk of developing neurodegenerative disorders.^31^

The higher prevalence of MCI within the LATE group supports the hypothesis that LATE may correspond to earlier and less severe stages of cognitive impairment.^1,5^ Confirmed cases of LATE typically exhibit a memory loss profile described as temporo-limbic amnesia or “hippocampal-type” amnesia,^32^ reflecting the overlapping topographic pattern of pathology in AD and LATE. In our study, patients classified as having probable LATE showed global cognition scores similar to those of the AD group and slightly higher scores in memory screening tests, such as Three-Objects-Three-Places. When considering a more specific memory test such as the FCSRT, participants with LATE and AD had similar scores. In addition to the amnestic profile, patients with autopsy-confirmed AD and LATE commonly have evidence of mild semantic memory deficits, often detected as a slight relative decrease in performance on measures of category fluency (e.g., generation of animal names).^4,31^ In our study, both patients with AD and LATE demonstrated overlapping scores on categorical and phonemic verbal fluency tests. Notably, participants with LATE did not exhibit lower scores on executive function tests, supporting the predominantly amnestic nature of this disease entity. Overall, these findings indicate that our identified LATE group exhibited predominantly mild memory impairment compared with the No-AD/No-LATE group, presenting a clinical profile that closely resembles typical features seen in AD.

Neuropathologically, LATE is characterized by neurodegenerative changes and astrogliosis in the amygdala, hippocampus, and entorhinal cortex, with pronounced atrophy in these regions.^5,11^ LATE has also been associated with atrophy in the inferior frontal, anterior temporal, and insular cortices.^12,13^ Although neuropathologic confirmation was not available in our study, MRI analyses revealed significantly reduced amygdalar and hippocampal volumes, as well as reduced cortical thickness in key regions associated with TDP-43 pathology, in patients with LATE and AD/LATE compared with the AD group, consistent with previous clinicopathological findings.^15^ Moreover, all 3 groups exhibited lower volumes and reduced cortical thicknesses relative to the No-AD/No-LATE group, further supporting our results. Of interest, regions known to be linked to AD pathology, such as the precuneus and posterior cingulate cortex, did not exhibit significant differences among AD, LATE, and AD/LATE groups. These findings suggest that the AAS, when integrated with AD biomarkers, may be useful for identifying limbic-predominant neurodegeneration.

Longitudinal analysis demonstrated similar cognitive trajectories in the LATE group compared with the No-AD/No-LATE group, with both declining more slowly than AD and AD/LATE groups over a 30-month follow-up period. This finding supports the validity of our diagnostic approach and highlights the clinical importance of distinguishing LATE from AD, given that differences in cognitive trajectories have significant implications for prognosis and patient management. Previous reports support our finding in this study, indicating progression of LATE to be more gradual than that of other neurodegenerative diseases, particularly AD.^3,31,33^ The absence of significant differences in cognitive decline over time between the AD and AD/LATE groups warrants further investigation. Given that our classification relied on a visual scale of amygdalar atrophy without neuropathologic confirmation, it is plausible that cognitive trajectories in the AD/LATE group were primarily driven by AD pathology rather than TDP-43. As previously noted, AD pathology can also contribute to amygdalar atrophy.^17^ In recognition of that, we believe that with our approach, co-pathology cases should be considered as possible, and not probable, AD/LATE. In such instances, a comprehensive evaluation becomes even more crucial, and we view the AAS as a tool that can be used purely for diagnostic support, to be integrated with other current and future methods capable of providing quantitative estimates of the predominant individual pathologic contributions.^34^

The use of biomarkers in CU individuals remains a topic of ongoing debate because current evidence is insufficient to support their routine use in clinical practice. Given that the primary objective of this study was to assess the added value of the AAS in the diagnostic workup of patients referred to a memory clinic, our main analysis included CU and cognitively impaired participants. Early identification of individuals at risk of developing cognitive impairment or dementia—potentially enabling preventive interventions—represents a key priority for future memory clinics.^35^ However, we believe that the most immediate and practical application of AAS may lie in assisting clinicians by providing diagnostic and prognostic insights for cognitively impaired patients seeking medical attention. To better assess the real-world utility of AAS in clinical decision making, we conducted sensitivity analyses excluding CU individuals. These analyses confirmed the diagnostic and prognostic relevance of AAS within the cognitively impaired population.

Our findings emphasize the urgent need for the development of in vivo biomarkers specific to limbic-predominant TDP-43 pathology.^36^ Neuroimaging features, such as fluorodeoxyglucose (FDG)-PET, have been investigated as potential indicators of LATE in vivo.^37-39^ For example, a temporo-limbic FDG-PET signature has recently been proposed as indicative of LATE.^37^ Antemortem FDG-PET patterns validated in LATE-NC (N = 7) and AD groups confirmed by autopsy were subsequently applied to stratify an independent cohort with clinically diagnosed AD dementia, identifying cases with LATE-NC–like profiles. Similar to our findings, these cases exhibited a predominant memory profile and a generally slower disease course. AAS is even more widely accessible because MRI is currently recommended as a first-level screening procedure for the diagnosis of cognitive disorders,^40^ making it particularly appealing for routine clinical practice. It is important to note that it could be applied without significant additional costs because it uses scans already performed in standard care.

Several limitations of this study warrant consideration. First, the retrospective design and single-center setting limit the generalizability of our findings to other populations. Because the AAS was applied retrospectively to a real-world memory clinic cohort, where investigations were determined by routine clinical practice or other ongoing research protocols rather than predefined by study design, a risk of selection bias is inherent. For example, the proportions of etiologic groups identified in our study may not reflect those in the general population: patients with LATE, who are typically older, may have been less likely to undergo AD biomarker assessments, while those with AD/LATE, being more severely impaired, may similarly have had a lower probability of undergoing such investigations. These issues not only affect the generalizability of our findings but may also affect their internal validity. For these reasons, our results should be interpreted with caution, and future studies with larger and prospectively designed samples, together with harmonized biomarker assessments, will be needed to validate and extend these findings. Second, the relatively small sample size of the LATE group possibly lowered the statistical power for some comparisons. Third, other potential contributors to amygdalar atrophy, such as alpha-synuclein pathology or TDP-43 frontotemporal lobar degeneration,^18,41^ were not evaluated because of the lack of specific biomarker and lack of autoptic confirmation in our cohort.

While the precise role of AAS in the diagnostic workup of memory clinic patients remains to be validated in prospective, diverse, and independent cohorts, our findings suggest its added value.^19^ Future studies should aim to validate the utility of AAS in prospective, multicenter cohorts and investigate its integration with emerging biomarkers of TDP-43 limbic-predominant pathology. Longitudinal studies with extended follow-up periods are needed to better characterize the natural history of LATE and its interplay with coexisting AD and other pathologies. In addition, the increasing availability of blood-based biomarkers for AD may enhance the feasibility of implementing diagnostic approaches for LATE, particularly for older patients who may face contraindications to CSF-based biomarker analysis.^42^

In conclusion, cases identified as LATE through the integration of the AAS with established AD biomarkers exhibited predominantly mild amnestic cognitive impairment, significant limbic atrophy on MRI, and a relatively slow rate of cognitive decline over time. These features align with findings from previous autopsy-based studies. Our results suggest that the AAS, when combined with current AD biomarkers, could serve as a valuable, accessible tool for differentiating LATE from other neurodegenerative conditions. However, this proof-of-concept study requires prospective validation and neuropathologic confirmation to fully establish the clinical utility of the proposed approach.

## Glossary

AAS: amygdalar atrophy scale
Aβ: β-amyloid
AD: Alzheimer disease
AD/LATE: AD and LATE co-pathology
ADNC: AD neuropathologic change
CU: cognitively unimpaired
FDG: fluorodeoxyglucose
GMC: Geneva Memory Center
FCSRT: Free and Cued Selective Reminding Test
LATE: limbic-predominant age-related TDP-43 encephalopathy
LATE-NC: LATE neuropathologic change
LME: linear mixed-effects
MCI: mild cognitive impairment
MMSE: Mini-Mental State Examination
MTA: medial temporal atrophy
p-tau181: phosphorylated tau 181
SCD: subjective cognitive decline
TDP-43: transactive response DNA-binding protein 43 kDa
TIV: total intracranial volume
